# High-dose dexamethasone and prolonged infusion time prevent oxaliplatin-related hypersensitivity reactions in patients with metastatic colorectal cancer

**DOI:** 10.1007/s10147-026-03037-8

**Published:** 2026-05-27

**Authors:** Koichiro Yoshino, Hiroki Osumi, Akira Ooki, Shohei Udagawa, Mikako Tamba, Shota Fukuoka, Takeru Wakatsuki, Mariko Ogura, Keisho Chin, Kensei Yamaguchi, Eiji Shinozaki

**Affiliations:** Department of Gastroenterological Chemotherapy, Cancer Institute Hospital of Japanese Foundation for Cancer Research, 3-8-31 Ariake, Koto-ku, Tokyo, 135-8550 Japan

**Keywords:** Oxaliplatin, Hypersensitivity reaction, Prevention protocol, Metastatic colorectal cancer

## Abstract

**Background:**

Oxaliplatin (OXA), a key cytotoxic agent used in the treatment of gastrointestinal cancers, is frequently discontinued due to hypersensitivity reactions (HSRs). We developed a protocol to mitigate HSRs and assessed its clinical efficacy.

**Methods:**

We retrospectively analyzed HSRs in patients with metastatic colorectal cancer (mCRC) who were treated with OXA-containing regimens between 2005 and 2019. We evaluated the clinical utility of our HSR prevention protocol in patients who experienced grade 1–3 HSRs following OXA-based chemotherapy. The protocol involved increasing dexamethasone to 16.5 mg/body and extending the OXA infusion time from 2 to 4 h.

**Results:**

Among 205 patients, 105 (51.2%) experienced grade 1, 95 (46.3%) grade 2, and 5 (2.4%) grade 3 HSRs. Most patients (87.8%) received either the FOLFOX4 or modified FOLFOX6 regimen. Patients underwent a median of 11 OXA cycles before their initial HSR. OXA was successfully reintroduced in 108 patients (52.7%) using the HSR prevention protocol. The median OXA-free interval was 23 days and the median progression-free survival after reintroduction was 6.4 months (95% confidence interval [CI]: 4.6–8.2). Reasons for discontinuation included a second HSR in 97 patients (47.3%), disease progression in 74 (36.1%), and chemotherapy-induced peripheral neuropathy (CIPN) in 15 (7.3%). Forty-six patients (22.4%) experienced an HSR during the initial prevention protocol. Only six patients (2.9%) experienced grade 3 HSRs and no grade 4 or 5 events were observed.

**Conclusion:**

High-dose dexamethasone and prolonged infusion time may enable OXA reintroduction, providing an alternative to permanent discontinuation in mCRC patients with prior HSRs.

**Registry number:**

2024-GB-073 (retrospectively registered).

**Supplementary Information:**

The online version contains supplementary material available at 10.1007/s10147-026-03037-8.

## Introduction

Colorectal cancer (CRC) is the fourth leading cause of cancer-related mortality worldwide, accounting for approximately 900,000 deaths annually. It also represents about 10% of all diagnosed cancers and cancer-related deaths globally. CRC is the second most common cancer in women and the third in men [1]. Although the prognosis for patients with metastatic colorectal cancer (mCRC) remains poor, it has improved in recent years due to advances in diagnostic techniques and treatment approaches, including surgery and chemotherapy.

Oxaliplatin (OXA) is a third-generation platinum-based cytotoxic agent. Similar to other platinum-based agents, such as cisplatin (CDDP) and carboplatin (CBDCA), OXA exerts its antitumor activity by inhibiting DNA synthesis through cross-linking with DNA bases [2]. Structurally, OXA differs from other platinum compounds in that it contains a 1,2-diaminocyclohexane group as its carrier ligand and an oxalate group as its leaving group [2]. OXA demonstrates strong antitumor activity against CRC cell lines and is considered a key drug in CRC treatment. Compared with CDDP, OXA is less nephrotoxic and less myelotoxic than CBDCA [3]. It is one of the standard therapies for postoperative adjuvant chemotherapy and first-line cytotoxic chemotherapy for mCRC [4], as recommended by several international guidelines for CRC treatment [5–7]. In Japan, OXA is also indicated for the treatment of metastatic pancreatic [8], gastric [9–12], small bowel [13], and esophageal cancers [14, 15].

The most common adverse events associated with OXA include hypersensitivity reactions (HSRs), chemotherapy-induced peripheral neuropathy (CIPN) [16], myelosuppression, and gastrointestinal symptoms. HSRs to platinum compounds are a well-recognized phenomenon [17]. As early as the 1950s, literature reported that exposure to platinum salts induced bronchial asthma in platinum refinery workers [18]. These reactions were first described for CDDP [19, 20], with similar reactions also reported for CBDCA [21, 22]. This type of toxicity has been sporadically noted in clinical trials evaluating the efficacy of OXA in chemotherapy and has been described in several case reports. Systematic reviews have shown prevalence rates of 10–18.9% for OXA, 5–20% for CDDP, and 1–44% for CBDCA. Allergic reactions are frequently cited as a reason for switching chemotherapy regimens [23].

The main symptoms of HSRs include facial and palmar flushing, pruritus and other cutaneous manifestations, respiratory symptoms such as dyspnea, gastrointestinal symptoms such as nausea and vomiting, and potentially life-threatening circulatory disturbances. Life-threatening reactions are reported to occur in approximately 1% of cases [23–25]. Severe HSRs requiring discontinuation of OXA may limit treatment options and reduce overall survival (OS).

Identifying risk factors for HSRs is important for predicting patient risk; however, some reports suggest that these reactions are unpredictable due to the variability in onset patterns and symptom presentation [26]. Reported risk factors include the number of OXA courses [27], cumulative OXA dose [28], oxaliplatin-free interval (OFI) [29], history of allergies, and female sex [30]. With appropriate supportive care to mitigate HSRs, OXA can be administered for longer durations, potentially improving survival outcomes.

Several protocols to prevent HSRs have been reported, with rechallenge success rates ranging from 28.6 to 89% [27, 28, 31–35]. These protocols commonly involve increasing corticosteroid doses or extending the infusion duration. Reported corticosteroid regimens include methylprednisolone at 1 mg/kg [32] [34] or 120 mg [33, 35], hydrocortisone at 500 mg [31], and dexamethasone at 20 mg [27, 28]. Extended infusion durations include increasing the administration time from 2 to 6 h [31] or gradually escalating the infusion rate to complete administration within 4 to 6 h [28, 33–35]. However, such protocols are often unsuitable for outpatient settings due to complex administration procedures and prolonged infusion times.

Based on previous reports and our clinical experience, we developed an HSR prevention protocol for OXA. This study aimed to evaluate the efficacy and safety of this protocol in patients with mCRC who experienced OXA-induced HSRs.

## Patients and methods

This single-institution retrospective study evaluated the efficacy and safety of a HSR prevention protocol in patients who experienced OXA-induced HSRs. The primary endpoint was the incidence of all grades of OXA-induced HSRs. Secondary endpoints included progression-free survival (PFS), overall survival (OS), overall response rate (ORR), disease control rate (DCR), and safety. We retrospectively enrolled patients with mCRC who were treated with OXA-containing chemotherapy regimens and developed OXA-induced HSRs between 2005 and 2019 at the Cancer Institute Hospital of the Japanese Foundation for Cancer Research in Tokyo, Japan. OXA-containing chemotherapy regimens included FOLFOX-based (leucovorin, fluorouracil, and OXA) and CapeOX-based (capecitabine and OXA) regimens.

### Ethics statement

This study was approved by the Institutional Review Board of the Japanese Foundation for Cancer Research, Tokyo, Japan (registry number 2024-GB-073). The study protocol was posted on the hospital website, and participants were given the opportunity to opt out; therefore, additional informed consent was not required. All procedures were conducted in accordance with the principles of the Declaration of Helsinki.

### Standard premedication protocol

Before experiencing the initial HSR, all patients received standard premedication consisting of palonosetron 0.75 mg/body administered over 15 min, dexamethasone 6.6 mg/body administered over 15 min, chlorpheniramine 5 mg/body administered over 5 min (when prescribed), and famotidine 20 mg/body administered over 5 min. Oxaliplatin was administered over 2 h at standard doses (85 mg/m^2^ for FOLFOX-based regimens and 130 mg/m^2^ for CapeOX-based regimens).

### HSR prevention protocol

**Fig. 1:**
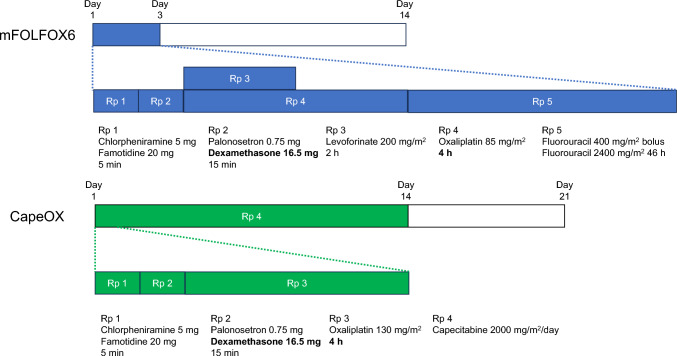
Hypersensitivity prevention protocol. The difference between the HSR prevention protocol and the usual regimen is that dexamethasone is increased from 6.6 mg to 16.5 mg/body, and the duration of OXA administration is extended from 2 to 4 h. *HSR* hypersensitivity reaction, *OXA* oxaliplatin, *Rp* recipe

The HSR prevention protocol included increasing the doses of dexamethasone to 16.5 mg/body, chlorpheniramine to 5 mg/body, and famotidine to 20 mg/body, and extending the OXA infusion time from 2 to 4 h (Fig. 1). For the FOLFOX-based regimen, premedication consisted of chlorpheniramine 5 mg/body and famotidine 20 mg/body administered over 5 min, followed by palonosetron 0.75 mg/body and dexamethasone 16.5 mg/body administered over 15 min. OXA (85 mg/m^2^), dissolved in 250 mL of 5% dextrose, was administered over 4 h. After completion of OXA administration, leucovorin (200 mg/m^2^) was administered over 2 h, followed by a rapid intravenous (IV) bolus of fluorouracil (400 mg/m^2^) and a continuous IV infusion of fluorouracil (2,400 mg/m^2^) over 46 h. For the CapeOX-based regimen, premedication included chlorpheniramine 5 mg/body and famotidine 20 mg/body administered over 5 min, followed by palonosetron 0.75 mg/body and dexamethasone 16.5 mg/body administered over 15 min. OXA (130 mg/m^2^), dissolved in 500 mL of 5% dextrose, was administered over 4 h. Capecitabine was administered at 2,000 mg/m^2^/day, beginning on the evening of the day of OXA administration.

### Data collection

Patient characteristics—including age, sex, body surface area (BSA), eosinophil counts, *KRAS* status, primary tumor site, and metastatic sites—number of OXA courses until HSR onset, cumulative OXA dose, OFI, initial HSR grade, treatment outcomes of the HSR prevention protocol, safety, and factors contributing to the protocol’s success—were assessed. The cumulative OXA dose was calculated as the sum of the product of the number of doses and the dose reduction rate. This may differ from the actual dose administered due to fractional adjustments or other clinical factors. HSR grades were recorded in electronic medical records and assessed using the Common Terminology Criteria for Adverse Events (CTCAE), version 4.0. Under CTCAE v4.0, the applicable system organ class is “immune system disorders,” and the corresponding preferred term is “allergic reaction.”

### Statistical analysis

PFS was defined as the time from protocol initiation to the first objective evidence of disease progression or death from any cause. OS was defined as the time from initiation of the HSR prevention protocol to the date of death. PFS and OS were estimated using the Kaplan–Meier method. Complete response (CR), partial response (PR), stable disease (SD), and progressive disease (PD) were defined according to the Response Evaluation Criteria in Solid Tumors, version 1.1. OFI was defined as the number of days from the onset of the initial HSR to the day the HSR prevention protocol regimen was administered. Logistic regression analysis was used to identify factors associated with second HSR. For continuous variables, median values were used as cut-off points in the exploratory analysis to ensure balanced group sizes and adequate statistical power. Receiver operating characteristic (ROC) curve analysis was performed to determine optimal cut-off values for key predictive factors. The area under the curve (AUC) and optimal cut-off points were calculated using the Youden index. Variables with p-values < 0.05 in univariate analysis or those with marginal significance (p < 0.10) and clinical relevance were included in multivariate analysis. Variables with p-values < 0.05 were considered statistically significant. All statistical analyses were performed using EZR software version 1.55 (Saitama Medical Center, Jichi Medical University, Saitama, Japan), which is based on R and R Commander [36].

## Results

### Patient characteristics

A total of 205 patients were enrolled between August 2005 and January 2021 (Table 1). The median age was 60 years (range: 27–80), and 109 patients (53.2%) were female. Seventy-six patients (37.6%) had a history of drug and/or food allergies. Eastern Cooperative Oncology Group performance status scores were 0 in 153 patients (74.6%), 1 in 50 patients (24.4%), and 2 in 2 patients (1.0%). The median eosinophil count at initial HSR onset was 82.8/μL (range: 0–1070). Additionally, 180 patients (87.8%) received FOLFOX-based chemotherapy, while 25 patients (12.2%) received CapeOX-based chemotherapy. Most patients received OXA as either first-line (n = 140, 68.3%) or second-line (n = 41, 20.0%) treatment for mCRC. There were no significant differences in baseline characteristics between the successful and unsuccessful rechallenge groups.

**Table 1:** Patient Characteristics

Characteristics	No. of Patients (%) N = 205, N (%)	Success of rechallenge N = 108, N (%)	Failure in rechallenge N = 97, N (%)	p value
Age at hyper sensitivity reaction				
Median [range], y	60 [27–80]	60 [27–77]	61 [36–80]	0.81
Sex				0.21
Male	96 (46.8)	46 (42.6)	50 (51.5)	
Female	109 (53.2)	62 (57.4)	47 (48.5)	
Body Surface Area				
Median [range], m^2^	1.60 [1.21–2.15]	1.60 [1.21–2.15]	1.60 [1.26–1.96]	0.74
History of Allergic drugs and foods				0.56
Present	76 (37.6)	43 (39.8)	34 (35.1)	
Absent	129 (62.4)	65 (60.2)	63 (64.9)	
Performance Status				0.26
0	153 (74.6)	79 (73.1)	74 (76.3)	
1	50 (24.4)	29 (26.9)	21 (21.6)	
2	2 (1.0)	0 (0)	2 (2.1)	
Treatment line				0.26
1st	140 (68.3)	75 (69.4)	65 (67.0)	
2nd	41 (20.0)	20 (18.5)	21 (21.6)	
3rd	14 (6.8)	5 (4.6)	9 (9.3)	
4th	7 (3.4)	5 (4.6)	2 (2.1)	
5th	3 (1.5)	3 (2.8)	0 (0)	
KRAS status				0.56
Wild type	81 (39.5)	48 (44.4)	33 (34.0)	
Mutant type	39 (19.0)	25 (23.1)	14 (14.4)	
Not evaluable	85 (41.5)	35 (32.4)	50 (51.5)	
Primary site				0.33
Rectum	84 (41.0)	49 (45.4)	35 (36.1)	
Colon	118 (57.6)	58 (53.7)	60 (61.9)	
Others (anal canal 2, A + T 1)	3 (1.5)	1 (0.9)	2 (2.1)	
Metastatic Organ				
Lung	104 (50.7)	56 (53.7)	48 (49.5)	0.78
Liver	89 (43.4)	54 (50.0)	35 (36.1)	0.049
Lymph nodes	69 (33.7)	40 (37.0)	29 (29.9)	0.3
Peritoneal	31 (15.1)	15 (13.9)	16 (16.5)	0.70
Ovary	15 (7.3)	7 (6.5)	8 (8.2)	0.79
Local	12 (5.9)	4 (3.7)	8 (8.2)	0.24
Others	25 (12.2)	12 (11.1)	13 (13.4)	0.83
Chemotherapy				0.045
FOLFOX	120 (58.5)	55 (50.9)	65 (67.0)	
FOLFOX + Bevacizumab	54 (26.3)	31 (28.7)	23 (23.7)	
FOLFOX + Cetuximab	1 (0.5)	1 (0.9)	0 (0)	
FOLFOX + Panitumumab	4 (2.0)	2 (1.9)	2 (2.1)	
FOLFOXIRI + Bevacizumab	1 (0.5)	1 (0.9)	0 (0)	
CapeOX	8 (3.9)	7 (6.5)	1 (1.0)	
CapeOX + Bevacizumab	17 (8.3)	11 (10.2)	6 (6.2)	
Eosinophil counts at initial HSR onset				
Median [range], /µL	82.8 [0–1070]	86.4 [0–1070]	75.2 [0–753]	0.09
Number of OXA courses at initial HSR				
Median [range]	11 [2–47]	11 [2–35]	11 [2–47]	0.90
Oxaliplatin dosage				
Cumulatvie OXA dose, mg	1443 [233–6100]	1500 [279–5156]	1412 [233–6100]	0.95
Cumulatvie OXA dose / BSA, mg/m2	901 [170–3315]	901 [170–3144]	899 [170–3315]	0.93
Cumulatvie OXA dose / time, mg/hr	716 [116–3050]	745 [139–2578]	696 [116–3050]	1.00
Oxaliplatin-free interval				
Median [range], day	23 [13–890]	22 [13–890]	24 [14–725]	0.37

*BSA* body surface area, *FOLFOX* leucovorin [LV] + 5-fluorouracil [5-FU] + oxaliplatin, *FOLFOXIRI* LV + 5-FU + oxaliplatin + irinotecan, *CapeOX* capecitabine + oxaliplatin

### Details of the initial HSR

**Fig. 2:**
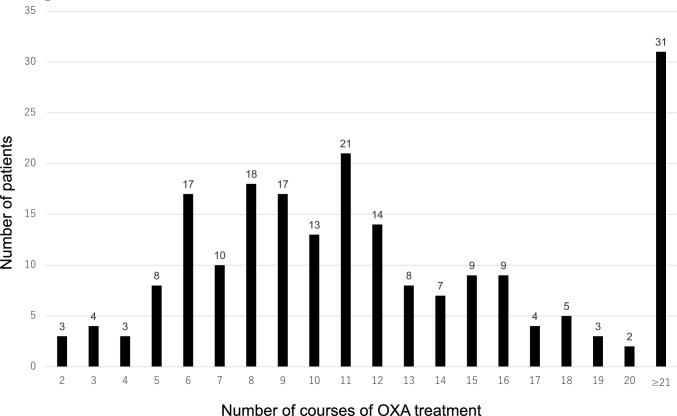
Number of courses and patients until HSR occurred. The numbers of OXA administrations and patients who experienced HSR from the start of treatment were recorded. *HSR* hypersensitivity reaction, *OXA* oxaliplatin

In the successful rechallenge group, the median number of OXA cycles before HSR onset, the median cumulative OXA dose, and the median dose per BSA were 11 (range: 2–35), 1,500 mg (range: 279–5,156) and 901 mg/m^2^ (range: 170–3,144) (Table 1, Fig. 2). In the failure rechallenge group, the corresponding figures were 11 (range: 2–47), 1412 mg (range: 233–6100), and 899 mg (range: 170–3315). The median OFI was 22 days (range: 13–890) in the successful rechallenge group and 24 days (range: 14–725) in the failure rechallenge group.

**Fig. 3:**
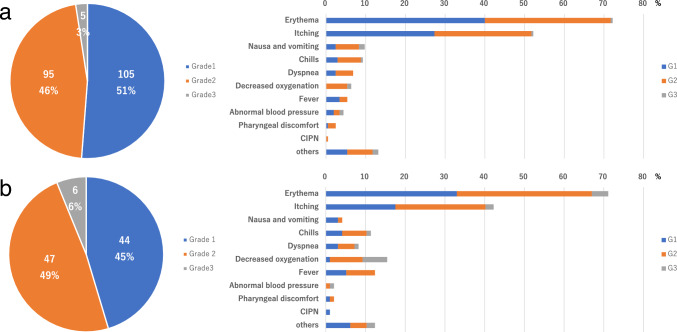
Details of the initial and second HSR. The pie chart shows the HSR grade, number of patients, and percentage. The bar graph shows HSR symptoms according to grade. Grade 3 symptoms related to circulatory and respiratory dynamics are common, and these symptoms increase with the second HSR. a. A total of 205 patients underwent initial HSR with OXA-based chemotherapy. b. A total of 97 patients with HSR were included in the HSR prevention protocol. *CIPN* chemotherapy induced peripheral neuropathy, *HSR* hypersensitivity reaction, *OXA* oxaliplatin

Grade 1 HSRs were the most frequent, occurring in 105 patients (51.2%), followed by grade 2 HSRs in 95 patients (46.3%) and grade 3 HSRs in 5 patients (2.4%) (Fig. 3a). Erythema occurred in 148 patients (72.2%), pruritus in 107 (52.2%), nausea and vomiting in 20 (9.8%), chills in 19 (9.3%), dyspnea in 14 (6.8%), decreased oxygen saturation in 13 (6.3%), fever in 11 (5.4%), abnormal blood pressure in 9 (4.4%), and pharyngeal discomfort in 5 (2.4%) (Fig. 3a).

### Details of the second HSR

A total of 205 patients received the HSR prevention protocol, among whom 97 (47.3%) experienced a second HSR. Forty-seven patients (22.9%) experienced an HSR on the same day the prevention protocol regimen was administered. The median time to the second HSR was 14 days (range: 0–910). Grade 1 HSRs occurred in 44 patients (45.4%), grade 2 in 47 (48.4%), and grade 3 in 6 (6.2%). No grade 4 or 5 HSRs were reported (Fig. 3b). Erythema occurred in 69 patients (71.1%), pruritus in 41 (42.3%), nausea and vomiting in 4 (4.1%), chills in 11 (11.3%), dyspnea in 8 (8.2%), decreased oxygen saturation in 15 (15.5%), fever in 12 (12.4%), abnormal blood pressure in 2 (2.0%), and pharyngeal discomfort in 2 (2.0%) (Fig. 3b). The proportion of patients experiencing chills, fever, and decreased oxygen saturation increased compared with the initial HSR.

### Reasons for discontinuing the HSR prevention protocol

**Fig. 4:**
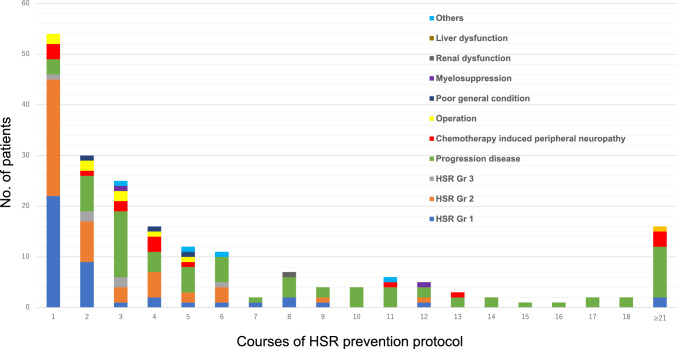
Reasons for discontinuing the HSR prevention protocol. Reasons for discontinuing HSR prevention protocols at frequency. A lower frequency resulted in a higher HSR percentage, whereas a higher frequency resulted in a higher PD percentage. *CIPN* chemotherapy induced peripheral neuropathy, *HSR* hypersensitivity reaction, *PD* progression disease

Among the patients who discontinued treatment, 44 (21.5%) experienced grade 1 HSRs, 47 (22.9%) experienced grade 2, and 6 (2.9%) experienced grade 3. In addition, 74 patients (36.1%) discontinued due to disease progression, and 15 (7.3%) due to CIPN (Table 2). Fifty-four patients discontinued at the start of the protocol, of whom 46 experienced an HSR (Fig. 4). The median number of protocol cycles was 3 (range: 1–40).

**Table 2:** Reasons for discontinuing HSR prevention protocol

Reasons for discontinuation	No. of patient total (%)
Chemotherapy induced peripheral neuropathy	15 (7.3)
2nd HSR Gr 1	44 (21.5)
2nd HSR Gr 2	47 (22.9)
2nd HSR Gr 3	6 (2.9)
Operation	8 (3.9)
Progression disease	74 (36.1)
Poor general condition	3 (1.5)
Myelosuppression	2 (1.0)
Renal dysfunction	1 (0.5)
Liver dysfunction	1 (0.5)
Others	4 (2.0)
Total	205 (100)

*HSR* hypersensitivity reaction

### Logistic regression analysis for risk factors of second HSR

ROC curve analysis was performed to determine optimal cut-off values for continuous variables. (Supplementary Figure S1, Supplementary Table S1).

Univariate logistic regression analysis showed that receiving the CapeOX-based regimen (odds ratio: 0.39; 95% confidence interval [CI]: 0.16–0.98; p = 0.04) and having an OFI ≥ 15 days (odds ratio: 3.44; 95% CI: 1.22–9.71; p = 0.02) were associated with the incidence of a second hypersensitivity reaction (HSR). Variables with marginal significance included eosinophil count (OR: 0.53; 95% CI: 0.27–1.04; p = 0.06), cumulative dose (OR: 0.57; 95% CI: 0.31–1.06; p = 0.07), and number of cycles (OR: 1.86; 95% CI: 0.94–3.68; p = 0.08). Eosinophil count < 47.6/μL at initial HSR (OR: 0.49; 95% CI: 0.25–0.99; p = 0.048), 5-FU-based regimen (vs. capecitabine-based; OR: 0.35; 95% CI: 0.12–0.97; p = 0.044), and OFI ≥ 15 days (OR: 3.13; 95% CI: 1.05–9.28; p = 0.04) remained independently significant in multivariate analysis (Table 3).

**Table 3:** Logistic regression analysis for risk factors of 2nd HSR

Factor	OR	Lower 95% CI	Upper 95% CI	p value
Uunivariate analysis				
Age (< 60* or ≥ 60 years)	1.26	0.73	2.19	0.41
Sex (Male* or Female)	0.70	0.40	1.21	0.20
BSA (< 1.48* or ≥ 1.48 m2)	1.21	0.66	2.24	0.54
Allergic history (Absent* or Present)	0.82	0.46	1.44	0.48
Eosinophil count (< 47.6* or ≥ 47.6)	0.53	0.27	1.04	0.06
Fluoropyrimidine (5-FU* or Capecitabine)	0.39	0.16	0.98	0.04
Cumulative dose (< 1969* or ≥ 1969 mg)	0.57	0.31	1.06	0.07
Cumulative dose / BSA (< 1323* or ≥ 1323 mg/m2)	0.69	0.36	1.30	0.25
Cumulative dose / time (< 984* or ≥ 984, mg/hr)	0.69	0.38	1.27	0.24
Number of courses (< 8* or ≥ 8 courses)	1.86	0.94	3.68	0.08
Oxalipratin free interval (< 15* or ≥ 15 days)	3.44	1.22	9.71	0.02
Initial HSR (Grade 1* or Grade 2,3)	1.14	0.66	1.98	0.64
Multivariate analysis				
Eosinophil count (< 47.6* or ≥ 47.6)	0.49	0.25	0.99	0.048
Fluoropyrimidine (5-FU* or Capecitabine)	0.35	0.12	0.97	0.044
Cumulative dose (< 1969* or ≥ 1969 mg)	0.82	0.40	1.68	0.59
Courses (< 8* or ≥ 8 courses)	1.49	0.65	3.42	0.35
Oxalipratin free interval (< 15* or ≥ 15 days)	3.13	1.05	9.28	0.040

*HSR* hypersensitivity reaction, *PFS* progression free survival, *OR* odds ratio, *CI* confidencial interval, *BSA* body surface area

^*^referrence

### Efficacy of the HSR prevention protocol

**Fig. 5:**
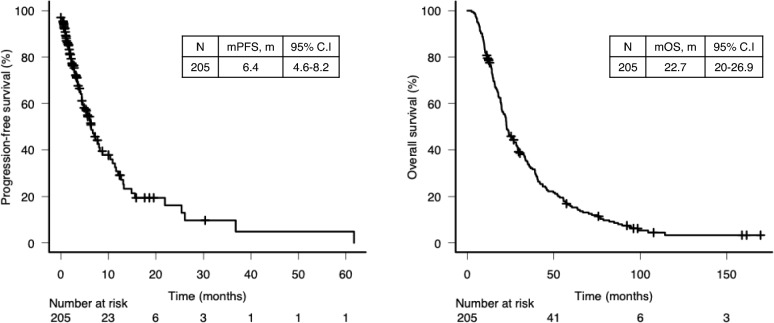
PFS and OS in patients with metastatic colorectal cancer receiving HSR prevention protocol. A total of 205 patients underwent the HSR prevention protocol. The median PFS was 6.4 months (95% CI 4.6–8.2 months). The median OS was 22.7 months (95% CI 20–26.9 months). HSR, hypersensitivity reaction; PFS, progression-free survival; OS, overall survival

A total of 108 patients (52.4%) were successfully rechallenged with OXA using the HSR prevention protocol. The ORR was 54.2%, comprising CR in 0.5%, PR in 53.7%, SD in 42.9%, and PD in 2.9%. The DCR was 97.1%. The median PFS was 6.4 months (95% CI: 4.8–8.2) and the median OS was 22.7 months (95% CI: 20.0–26.9) (Fig. 5).

**Fig. 6:**
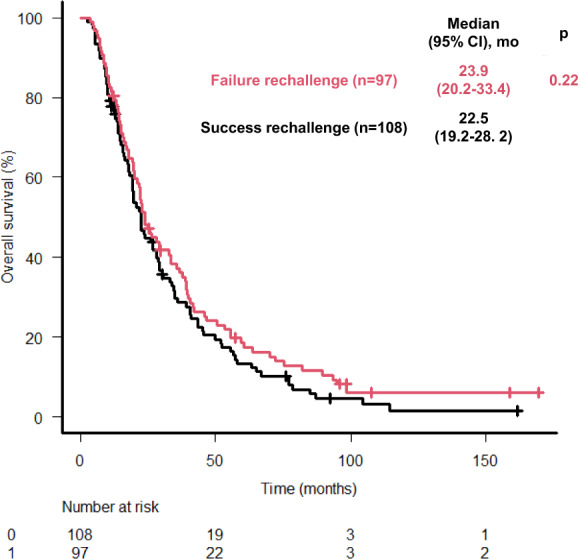
OS with or without a second HSR. The median overall survival was 23.9 months in patients in the re-challenge failure group (n = 97) and 22.5 months in patients in the re-challenge success group (n = 108). *OS* overall survival, *HSR* hypersensitivity reaction

OS was compared between patients who successfully continued oxaliplatin without second HSR and those who developed second HSR. The median OS was 22.5 months (95% CI: 19.2–28.2) in the successful rechallenge group and 23.9 months (95% CI: 20.2–33.4) in the failure rechallenge group (p = 0.22) (Fig. 6). There was no significant difference in OS between the two groups.

## Discussion

In this study, we evaluated the efficacy and safety of a hypersensitivity reaction prevention protocol combining high-dose dexamethasone (16.5 mg/body) and prolonged infusion time (4 h) in 205 patients with metastatic colorectal cancer who experienced grade 1–3 oxaliplatin-induced hypersensitivity reactions. Our results demonstrated that 108 patients (52.7%) were successfully rechallenged with oxaliplatin, achieving a median progression-free survival of 6.4 months and median overall survival of 22.7 months. Importantly, only 6 patients (2.9%) experienced grade 3 s hypersensitivity reactions, with no grade 4 or higher severe reactions observed. Furthermore, we identified eosinophil counts < 47.6/µL and CapeOX-based regimen as a protective factor, while oxaliplatin-free interval ≥ 15 days was identified as a risk factor for second hypersensitivity reactions. These findings provide a practical alternative to permanent oxaliplatin discontinuation due to hypersensitivity reactions.

Several previous studies have reported the efficacy of rechallenge protocols for oxaliplatin-induced hypersensitivity reactions (Table 4). Compared with these previous reports, our study has several notable advantages. First, our study included a larger patient cohort (n = 205) Second, our simplified protocol is feasible in an outpatient setting. Third, our protocol is more straightforward and easier to implement in routine clinical practice, requiring fewer steps and less complex preparation than traditional 12-step desensitization methods. Most importantly, our study is unique in providing comprehensive survival data. To the best of our knowledge, this is the largest study to evaluate the safety and efficacy of an HSR prevention protocol in patients with mCRC who experienced grade 1–3 HSR after receiving OXA-based chemotherapy.

**Table 4:** Previous and our study

References	First author	Year	Patient number	Success rate	Administration method	Premedication
[27]	Kidera Y	2011	181 patients (Prevention group: 100)	93/100 (93%)*	Standard administration	Dexamethasone 20 mg, Diphenhidramine 50 mg, Famotidine 20 mg, Granisetron 3 mg
[28]	Okayama T	2015	162 patients (HSR: 28, rechallenge: 7)	5/7 (71%)	Premedication-1 Stepup-dosing (4 step / 1 h) Premedication-2 Prolonged infusion time (4 h)	Premedication-1: Dexamethasone 20 mg, Diphenhydramine 50 mg, Domperidone 10 mg, Famotidine 20 mg, Premedication-2: Hydrocortisone 100 mg, Granisetron 3 mg
[31]	Yanai T	2012	623 patients (HSR: 126, rechallenge: 99)	55/99 (56%)	STEP 1: Standard premedication STEP 2: Prolonged infusion time (6 h) STEP 3: Add subcutaneous epinephrine	STEP 1: Hydrocortisone 100 mg, Chlorpheniramine 10 mg, Ranitidine 50 mg STEP 2: Hydrocortisone 500 mg STEP 3: STEP 2 + Subcutaneous Epinephrine 0.3 mg
[32]	Syrigou EI	2009	215 patients (HSR: 52, rechallenge: 32)	12/32 (38%)	Standard administration	Methylprednisone 1 mg/kg (maximum 60 mg), Ranitidine 150 mg, Cetirizine 10 mg at 13, 7, and 1 h before oxaliplatin administration
[33]	Botsen D	2019	431 patients (HSR: 24, rechallenge: 7)	4/7 (57%)	Step-up dosing (6 steps / 30 min) Prolonged infusion time (3 h)	Methylprednisolone 120 mg, Dexchlorpheniramine 2 mg taken 48 and 24 h and 30 min before
[34]	Cortijo-Cascajares S	2013	21 patients	17/21 (81%)	Step-up dosing (14 steps / 10 min) Prolonged infusion time (3,4 h)	Colticosteroid 1 mg/kg, Ranitidine 300 mg, Cetirizine 20 mg, Montelukast 10 mg, administered the night before and 30 min before oxaliplatin administration
[35]	Rassy E	2023	54 patients	41/54 (76%)	Step-up dosing (7 steps / 30 min) Prolonged infusion time (3,4 h)	Methylprednisolone 120 mg, Dexchlorpheniramine 5 mg, Levocetirizine 5 mg 12 h and 4 h before oxaliplatin administration
	Yoshino K	2025	205 patients	108/205 (53%)	Prolonged infusion time (4 h)	Dexamethasone 16.5 mg, Palonosetron 0.75 mg, Chlorpheniramine 5 mg, Famotidine 20 mg

*HSR* hypersensitivity reaction

*Success rate in patients who have not undergone HSR

The mechanisms underlying OXA-induced HSRs remain incompletely understood. These reactions are thought to primarily involve type I hypersensitivity, also referred to as immediate hypersensitivity, which occurs within minutes of exposure [37]. This response is triggered by immunoglobulin E (IgE) antibodies that bind to the surface of mast cells and basophils. Upon re-exposure to the antigen, the IgE antibodies cross-link, leading to the release of intracellular granules (degranulation) [37]. These granules release large quantities of inflammatory mediators—such as histamine, serotonin, and heparin—causing rapid vasodilation and increased vascular permeability. The resulting plasma leakage from blood vessels can lead to hypotension, urticaria, and edema [37].

In this study, 108 patients (52.4%) were successfully rechallenged with OXA using the HSR prevention protocol. Previous reports have indicated that increasing the doses of dexamethasone and antihistamines from the sixth cycle of modified FOLFOX6 treatment significantly reduced the incidence of OXA-related HSRs [27]. While this strategy appears promising in suppressing OXA-related HSRs, increasing the dose of prophylactic agents such as corticosteroids for all patients may pose risks due to the potential for severe adverse effects.

Other reports have also shown that lowering the infusion rate and using premedication with corticosteroids and histamine receptor antagonists can enable successful re-administration of platinum agents. In a recent retrospective study by O’Ceabhaill et al., 777 patients with relapsed ovarian, fallopian tube, or primary peritoneal cancer were retreated with carboplatin [38]. The data indicated that gradually increasing the infusion duration to 3 h during carboplatin rechallenge, combined with appropriate premedication, may reduce the incidence of HSRs compared with the standard 30-min infusion [38]. Another study reported that continuous 6 h infusion of OXA may reduce the risk of hypersensitivity. In one trial, only 1 of 100 patients (1%) treated with OXA as a 6 h infusion added to chronomodulated 5-fluorouracil (5-FU) and folinic acid experienced an HSR as part of first-line therapy for advanced colorectal cancer [39]. Furthermore, no HSRs were observed in 151 patients who received 1,087 courses of constant-rate OXA infusion and in 491 patients who received 3,106 courses of chronomodulated OXA infusion [40]. Notably, five patients who developed HSRs during 2 h infusions of OXA did not experience symptoms when re-exposed to 6 h infusions.

The mechanism underlying this effect remains unclear; however, it is hypothesized that prolonged infusion reduces the drug’s maximum plasma concentration. In addition to the role of premedication, a rechallenge protocol involving prolonged OXA infusion for prophylaxis has been reported. According to Maindrault-Goebel et al., the duration of OXA infusion was extended from 2 to 6 h in five patients who had developed laryngospasm in response to OXA, and no recurrence of symptoms was observed [41]. Although the precise mechanism by which prolonged OXA infusion suppresses HSRs is unknown, it is assumed that slower administration may lower peak plasma levels.

In this study, the use of fluoropyrimidines—particularly 5-FU more than capecitabine—and an OFI longer than 15 days were identified as risk factors for a second HSR. Two previous reports investigated HSRs in CapeOX-based regimens [28, 42]; however, neither study found CapeOX to be a statistically significant risk factor. It is possible that HSRs were less likely to occur due to the lower number of treatment cycles compared with FOLFOX-based regimens. Additionally, although OFI has been discussed in the context of stop-and-go strategies [29, 43, 44], it has rarely been evaluated in the setting of HSR prevention protocols.

Our analysis identified several factors associated with rechallenge failure: low eosinophil count (< 47.6/μL) at initial HSR, prolonged OFI (≥ 15 days), particularly in stop-and-go strategies, and use of 5-FU-based regimens compared with capecitabine-based regimens. Patients with these risk factors may benefit from direct transition to alternative therapies rather than rechallenge attempts.

OS was similar between patients with and without second HSR (22.5 vs 23.9 months, p = 0.22), demonstrating that rechallenge attempts do not adversely affect long-term outcomes even when second HSR occurs. This finding underscores the clinical value of our prevention protocol. More than half of patients (52.7%) successfully continued oxaliplatin without experiencing a second HSR, and even among those who developed a second HSR, additional treatment cycles (median: 3 cycles) were administered before discontinuation. These additional cycles may have contributed to disease control and survival benefit. Furthermore, the low incidence of severe reactions during rechallenge (grade 3: 6.2%; no grade 4–5 events) supports the safety and feasibility of this approach. Although no significant difference was observed in this cohort, it may serve as a treatment option.

Based on the results of our study, we recommend the following treatment strategies for OXA-related HSRs: If grade 1 or 2 HSRs occur—excluding those with dyspnea—OXA-containing chemotherapy may be continued using a rechallenge protocol [31]. However, in cases of grade 2 or 3 HSRs involving cardiovascular or respiratory symptoms, the decision to continue or discontinue OXA-containing treatment should be made with caution [31]. If subsequent chemotherapies, such as irinotecan or anti-epidermal growth factor receptor antibodies, remain active options, we recommend switching to these agents or administering continuous infusion of 5-FU/leucovorin without OXA [31]. Particular caution should be exercised regarding the duration of OXA reintroduction in stop-and-go strategies [45], as prolonged reintroduction intervals may increase the risk of a second HSR.

Maintaining uninterrupted use of key drugs, when possible, may contribute to prolonged overall survival. Therefore, minimizing adverse effects is essential to achieving successful chemotherapy outcomes, and a comprehensive understanding of each drug’s safety and efficacy profile is required. The accuracy of adverse event monitoring should be enhanced through a multidisciplinary approach involving physicians, nurses, and pharmacists. Ongoing monitoring of side effects supports early detection and prevention, preserves quality of life, and contributes to improved treatment efficacy. When these two factors are present, caution and careful consideration are required when re-administering OXA due to the possibility of HSR.

This study has several limitations. Although it was retrospective in design, the sample size was relatively small, and definitive conclusions could not be drawn. Cutoff values for continuous variables were determined using ROC analysis within the same dataset, which carries a risk of overfitting. External validation is needed to confirm the generalizability of these thresholds. Nevertheless, the findings may still be valuable in informing everyday clinical practice.

In conclusion, the success rate of OXA reintroduction using the HSR prevention protocol was comparable to those reported in previous studies addressing manageable adverse events. This protocol offers a viable alternative to the permanent discontinuation of OXA in patients who develop HSRs.

## Supplementary Information

Below is the link to the electronic supplementary material.

Supplementary file1 (PPTX 117 KB)

Supplementary file2 (DOCX 23 KB)

## Data Availability

The datasets generated and/or analyzed during the current study are not publicly available but are available from the corresponding author upon reasonable request.
