# Supplementary material for: High-dose dexamethasone and prolonged infusion time prevent oxaliplatin-related hypersensitivity reactions in patients with metastatic colorectal cancer
Source: Int J Clin Oncol. 2026 May 27;31(7):1245–57. doi: 10.1007/s10147-026-03037-8 (PMC13303596; doi:10.1007/s10147-026-03037-8)
Supplement: Supplementary file 1 — Supplementary file1 (PPTX 117 KB) [file 10147_2026_3037_MOESM1_ESM.pptx]

## Slide 1
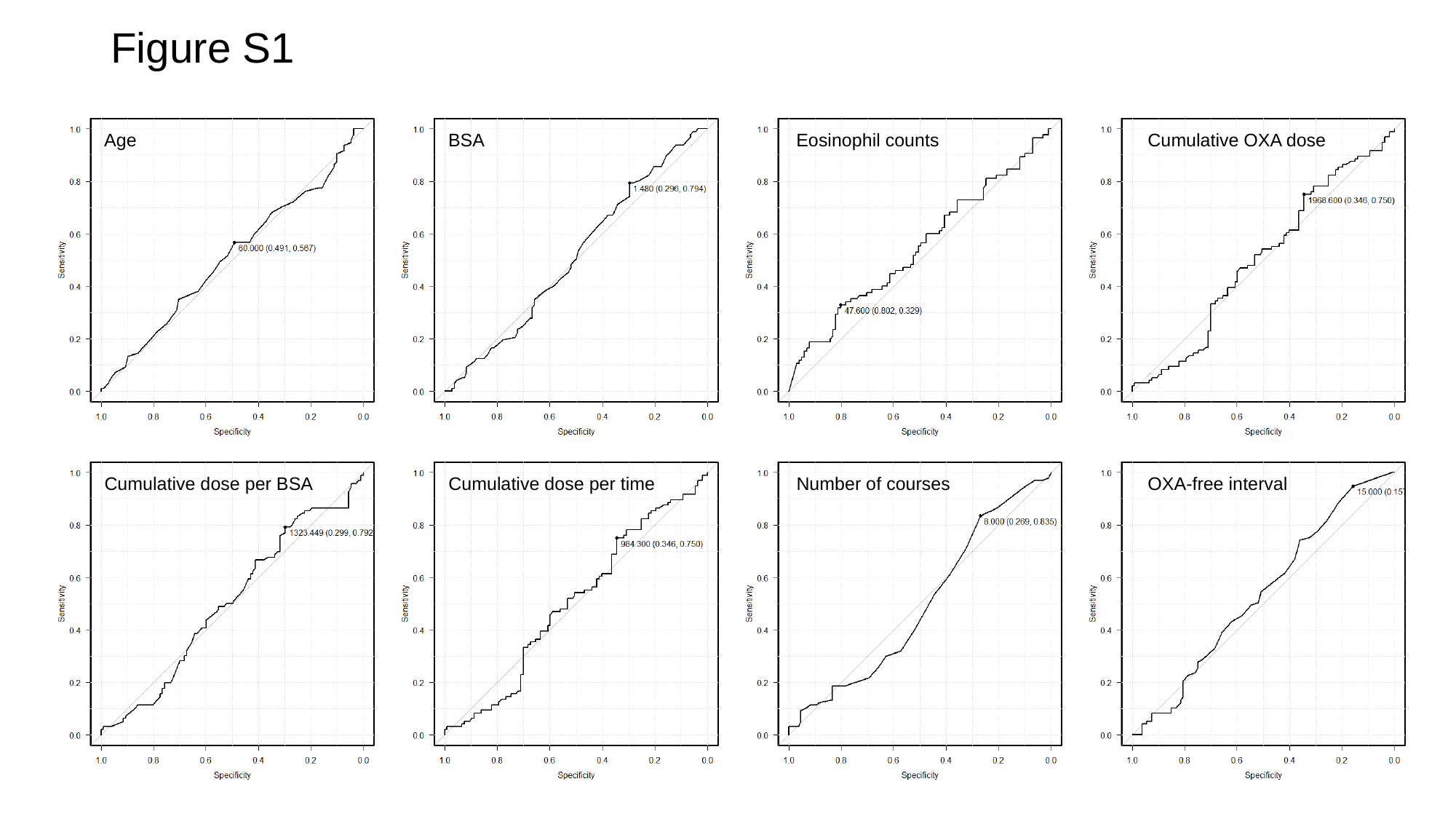

Figure S1
Age
BSA
Eosinophil counts
Cumulative OXA dose
OXA-free interval
Number of courses
Cumulative dose per BSA
Cumulative dose per time
