# Supplementary material for: High-dose dexamethasone and prolonged infusion time prevent oxaliplatin-related hypersensitivity reactions in patients with metastatic colorectal cancer
Source: Int J Clin Oncol. 2026 May 27;31(7):1245–57. doi: 10.1007/s10147-026-03037-8 (PMC13303596; doi:10.1007/s10147-026-03037-8)
Supplement: Supplementary file 2 — Supplementary file2 (DOCX 23 KB) [file 10147_2026_3037_MOESM2_ESM.docx]

| **Table S1. ROC Curves for Predicting Second HSR** | | | |  |  |
| --- | --- | --- | --- | --- | --- |
|  |  |  |  |  |  |
| **Variable** | **AUC** | **95% CI** | **Optimal Cut-off** | **Sensitivity** | **Specificity** |
| **Age** | 0.51 | 0.430-0.590 | 60 years | 56.70% | 49.10% |
| **BSA** | 0.51 | 0.434-0.593 | 1.48 m2 | 79.40% | 29.60% |
| **Eosinophil counts** | 0.55 | 0.461-0.630 | 47.6 /µL | 80.20% | 32.90% |
| **Cumulative dose** | 0.5 | 0.423-0.583 | 1968.6 mg | 75.00% | 34.60% |
| **Cumulative dose/BSA** | 0.51 | 0.425-0.585 | 1323.449 mg/m2 | 79.20% | 29.90% |
| **Cumulative dose/time** | 0.5 | 0.423-0.583 | 984.3 mg/hr | 75.00% | 34.60% |
| **Number of courses** | 0.5 | 0.415-0.575 | 8 | 83.50% | 26.90% |
| **OFI** | 0.54 | 0.458-0.616 | 15 days | 77.30% | 29.60% |
|  |  |  |  |  |  |
| abbreviation |  |  |  |  |  |
| HSR: hypersensitivity reaction |  |  |  |  |  |
| AUC: area under the curve |  |  |  |  |  |
| CI: confidential interval |  |  |  |  |  |
| BSA: body surface area |  |  |  |  |  |
| OFI: oxaliplatin-free interval |  |  |  |  |  |
